# Attitude towards the Promotion of Healthy Eating among Secondary School Teachers—Construction and Validation of a Questionnaire

**DOI:** 10.3390/nu14112271

**Published:** 2022-05-28

**Authors:** Almudena Garrido-Fernández, Francisca María García-Padilla, Juan Diego Ramos-Pichardo, Macarena Romero-Martín, Elena Sosa-Cordobés, Miriam Sánchez-Alcón

**Affiliations:** Department of Nursing, University of Huelva, 21071 Huelva, Spain; almudena.garrido@denf.uhu.es (A.G.-F.); fmgarcia@denf.uhu.es (F.M.G.-P.); juan.ramos@denf.uhu.es (J.D.R.-P.); macarena.romero@denf.uhu.es (M.R.-M.); miriam.sanchez@denf.es (M.S.-A.)

**Keywords:** healthy eating, attitudes, educators, secondary school

## Abstract

Health promotion activities in secondary schools are scarce and have little involvement of the teaching staff. Most often, activities are developed from the curriculum that appears in school materials, with little capacity for adaptation and innovation. The aim of this study was to construct and validate a tool to find out teachers’ attitudes towards activities to promote healthy eating in secondary schools. For this purpose, a descriptive study was conducted. The total sample of the study consisted of 200 teachers from secondary schools. Internal consistency was determined by Cronbach’s alpha coefficient globally and by dimension, and with the corrected item–test correlation. The construct validity of the questionnaire was assessed by means of an exploratory factor analysis, for which the principal components method with Varimax rotation was used. A Likert-type scale with nine items and four response options about attitude was designed. The exploratory factor analysis showed a nine-factor solution, of which two had eigenvalues greater than 1. These two factors explained 63.4% of the variance. The Cronbach’s alpha internal consistency index obtained for the global scale was 0.81, and 0.75 and 0.85 for each component. The results obtained with this structure confirmed an adequate reliability and validity of the questionnaire.

## 1. Introduction

Obesity and overweight are currently a major public health problem affecting the population globally, especially younger people. The World Health Organization (WHO) declared childhood obesity as one of the most serious public health challenges of the 21st century, before the advent of COVID-19 [1]. In 2016, 340 million children and adolescents were diagnosed as overweight or obese [2]. Institutions such as schools and secondary schools therefore need to be involved in promoting healthy eating.

WHO suggests putting in place school policies and programmes that promote healthy eating, as well as implementing specific actions for learning cooking and nutrition skills in schools. The organisation recommends involving both families and educators in this task [3]. Nutrition is one of the most relevant aspects of childhood and adolescent care due to its consequences on the overall development of the person [4,5]. Knowing the eating habits of adolescents and finding out whether secondary schools apply an adequate policy should be a priority [2,6].

The transition from primary to secondary school can lead to new habits in adolescents, as they have a greater ability to make unhealthy food choices more autonomously, thus minimizing parental authority in their eating behaviour [5,7,8].

Several studies show that content on healthy eating in secondary school curricula is scarce, and if it exists at all, it plays a negligible part in the curriculum [6,9,10,11,12,13,14]. In addition to few practical activities in schools, different degrees of non-compliance or ignorance of regulations on this issue have also been identified [15]. It is therefore essential to train secondary school teachers in the promotion of healthy eating among students, including the study and improvement of their attitudes towards these activities [6,16,17,18,19].

Improving knowledge, attitudes, and beliefs, as well as behavioural competence and self-efficacy about healthy eating, is often a precursor to behavioural changes [20]. Attitudes are known to influence and guide people’s behaviour. They are involved in determining an individual’s actions and are acquired through our individual and group life experience. They can be stimulated or changed, thus improving a person’s behaviour, and with it their lifestyles [21].

A previous study showed that health promotion activities in secondary schools in Huelva, are scarce and have little involvement of teachers, who are usually more focused on strictly academic activities [6,22]. Most often, activities are developed from the curriculum that appears in the school materials, with little capacity for adaptation and innovation. Therefore, it is necessary to have tools that reveal the attitudes of teachers towards the promotion and healthy eating in students, which will lead to interventions to improve teacher attitudes when necessary, and thus increase the involvement of teachers in the promotion of young people’s health.

The aim of this study was to construct and validate a tool to assess the attitudes of Spanish secondary school teachers towards healthy eating promotion activities in secondary schools.

## 2. Materials and Methods

The methodology used had two clearly differentiated phases: the construction of the questionnaire and its validation.

For the construction of the questionnaire, the indicators described as criteria for assessing secondary schools as centres for the promotion of healthy eating were taken as a reference [22]. A total of 56 indicators classified into eight categories were agreed upon through a Delphi group, one of these being teacher training, which included six indicators referring to attitudes towards the promotion of healthy eating. An initial questionnaire with nine items on teachers’ attitudes was constructed.

This initial questionnaire was subjected to a content validation process carried out by experts to assess the correspondence of the items in relation to the operational definition of the previously agreed indicators. As a group of experts, three people with the following profiles collaborated: a doctor in health and university lecturer specialized in food, nutrition, and dietetics; a university lecturer specialized in public health and health promotion; and a doctor in pedagogy and secondary education teacher.

The criteria of this group of experts were collected through an ordinal scale that included the following: strongly disagree; somewhat agree; fairly agree; and strongly agree. Aspects regarding the balance of items in relation to their level of relevance and the characteristics of their formulation (length, clarity, lack of double negatives, negative expressions, etc.) were also taken into account. After this evaluation, the list of nine items was maintained, and the items were formulated as statements. Answers were articulated according to the degree of agreement of the respondent with the statement contained in each item, through a Likert scale of response with four options: strongly disagree; somewhat agree; fairly agree; and strongly agree.

The questionnaire was piloted with 10 teachers from the target population to check their understanding of the questions and the functioning of the instrument. Their suggestions led to some corrections and the configuration of the final questionnaire for validation, keeping the nine items of the response scale described above.

For the validation phase, the sample consisted of 200 teachers from 22 secondary schools in the province of Huelva. The schools were randomly selected from a list provided by the Education Office. Subsequently, a visit to the secondary schools was arranged with the management teams by telephone and e-mail.

Once at the centre, the project was explained to the management team and/or guidance counsellor, and if they decided to participate, they were given the questionnaires in paper format. Approximately two weeks later, arrangements were made to collect the questionnaires at the centres.

Voluntary participation was requested from teachers in management positions, tutor teachers, school counsellors, and teachers of subjects whose official curriculum included content on the subject under study, as is the case of biology and physical education. Guidance counsellors were included, as they are responsible for the elaboration of the tutorial action plan, which is subsequently implemented in tutorial sessions and where food contents are included. A total of 200 secondary education teachers completed the questionnaire.

As for data analysis, internal consistency reliability was determined by calculating Cronbach’s alpha coefficient both globally and by dimension, and with the corrected item–test correlation. The construct validity of the questionnaire was assessed through an exploratory factor analysis, for which the principal components method with Varimax rotation was used. The SPSS software (version 26.0; IBM SPSS, Inc., Chicago, IL, USA) was used for data analysis.

This study was approved by the ethics committee of the University of Huelva and obtained the Authorisation of the Delegation of Education of Huelva. The Ministry of Education and Sport also established a collaboration agreement with the University of Huelva for the development of interventions in non-university public teaching centres of the Ministry of Education and Sport. The application of Organic Law 3/2018 of 5 December on the Protection of Personal Data and Guarantee of Digital Rights was guaranteed, not using such data for purposes other than those set out in the objectives and specific purposes of this study. In addition, the study was conducted following ethical principles set out in the Declaration of Helsinki. Participation in the study was free and voluntary. An informed consent model was delivered to the participants, ensuring the confidentiality of the data and their exclusive use for the aim of the study.

## 3. Results

### 3.1. Sociodemographic Results

The descriptive data of the sample can be seen in Table 1. The majority of the sample was between 30 and 40 years old. Notably, 57.5% were women and the vast majority (75.5%) had been teaching at the centre for less than 10 years. It should also be noted that 31.5% were part of the management team, 8.5% were counsellors, 37.5% were teachers of physical education or biology, and 23% of the sample consisted of tutors.

### 3.2. Descriptive Results

Table 2 shows the descriptive data at item level. All items had a mean of more than 3. Item 5 was the one with the highest mean. In all items of the questionnaire, all four response categories were used. The mean obtained for the total attitude was 29.21 (*SD* = 2.91).

### 3.3. Validity

The exploratory factor analysis showed a solution of nine factors, of which two presented eigenvalues greater than 1. These two factors explain 63.4% of the variance (Table 3). The factor weights of each item (Table 4) suggested a grouping of items 7, 8, and 9 in one dimension, and items 1 to 5 in another dimension, leaving item 6 with similar weights in both dimensions. Items 7 to 9 refer to attitudes towards participation in specific actions to promote healthy eating, so this dimension has been named proactivity (PRO), while items 1 to 5 refer to issues that generate concern among teachers in relation to student nutrition, so this dimension has been named concern (CON). Item 6, given its wording, has been included in the proactivity dimension, although its factorial weight is somewhat lower. The results per dimension showed a mean of 12.63 (*SD* = 2.91) for the proactivity dimension and 16.58 (*SD* = 3.04) for the concern dimension (Table 2).

### 3.4. Reliability

The Cronbach’s alpha internal consistency index obtained for the overall scale was 0.81, and for each component it was 0.75 and 0.85. Table 5 shows Cronbach’s alpha if each of the items is removed, both overall and for each of the dimensions.

Table 6 shows the corrected item–test correlations both for the overall scale and for each dimension. All items, except item 1 for the total, item 5 for the total, and the proactivity dimension, showed correlations above 0.40, which is the standard above which the correlation is considered satisfactory [23], with items 4 and 8 being the most highly correlated.

## 4. Discussion

The aim of this study was to develop a questionnaire to find out the attitudes of secondary school teachers towards the promotion of healthy eating in schools.

Childhood obesity remains a major health concern and a public health challenge. The problem has been aggravated by the impact of the COVID-19 pandemic on health systems, the economy, and children’s lifestyles [24]. The confinement imposed to prevent the spread of the virus has made it more difficult to maintain a healthy diet and has reduced the practice of physical activity [25]. Addressing childhood obesity requires a broad strategy involving the patient, family, school, community, and even government for policy changes [26]. For long-term results, interventions are needed that help create an environment and opportunities that make it easier for children to make healthy choices about nutrition and physical activity [27,28].

In this context, it is essential to have assessment scales for healthy lifestyles in children. In line with the scale resulting from this study, valid and reliable scales have been already designed to assess knowledge of healthy lifestyle [29], knowledge of nutrition [30], fruit and vegetable consumption [31], sedentary behaviours [32], habits of personal hygiene [33], oral health [34], or sun exposure [35] among children.

To date, no instruments that measure attitudes towards the promotion of healthy eating among secondary school teachers have been found. However, some that measure attitude or knowledge about eating and physical activity in adolescents and other indicators of healthy lifestyle habits have been identified [36,37,38,39,40,41]. There are also studies that explore knowledge, beliefs, and attitudes in primary and secondary school teachers [42] and in primary school students and families [43] using scales based on the KAP (Knowledge, Attitudes, Practices) model, but they study attitudes towards nutrition in general and not regarding health promotion, which is why the present study contributes with new data.

Regarding the factor structure of the instrument, the results showed two distinct dimensions: proactivity, made up of the four items related to active behaviour, and concern, which encompasses the other five items focused on the concern that the promotion of healthy eating may provoke in the informants. Previous studies have shown that a proactive attitude can lead to better health outcomes and encourage healthy lifestyles [44,45]. In addition, the concern and involvement of teachers has been identified as one of the main components of the implementation of health promotion in schools [46,47].

It should be noted that item 6 showed very similar factorial weights in both factors, but it was decided to include it in the proactivity dimension, since its wording (“I believe that teachers should get involved in promoting healthy eating among students”) makes it more suitable and representative of this dimension.

As for reliability, Cronbach’s alpha indicators and corrected item–test correlations indicated good internal consistency of the questionnaire, both overall and in each of the two dimensions identified in the factor analysis. Given the small size of the proposed questionnaire (nine items) and the small improvement in Cronbach’s alpha if some items were removed, it seems most reasonable to maintain the initially proposed structure of nine items in two dimensions.

Items 1 for the total scale and 5 for the concern dimension raise Cronbach’s alpha if they are removed, but the difference is minimal. As for the item–test correlations, items 1 for the total scale, 5 for the total scale, and the concern dimension, they do not reach the score of 0.40, which is indicated as ideal [23], but they do highlight the good internal consistency when done by dimensions, and they help to maintain a high Cronbach’s alpha, which is why it was decided to keep them in the final questionnaire.

Item 6 slightly raised Cronbach’s alpha in the proactivity dimension if the item was removed, a fact that we expected since, as mentioned when discussing the factor weights, this item was included in this dimension due to the small difference between dimensions and its better fit with the proactivity dimension. Moreover, in the overall scale, we can see how its removal would reduce internal consistency.

The selected sample comes from XXX and the province of XXX, so studies with a larger and more diverse sample would be necessary to verify the universality of the questionnaire.

The promotion of attitudes towards the adoption of healthy behaviours is important for the effective promotion of healthy eating [40,48,49]. In the present study, it can be seen that the general attitude of the studied educational community towards the promotion of healthy eating is positive, both in general and in terms of dimensions. As proposals for future interventions, it is worth highlighting the possibility offered by these results to identify health assets in schools and, therefore, to create relationships, provide resources, and establish interventions to promote healthy eating. On the other hand, given the current high influence of the Internet on children and adolescents, considered digital natives, web-based interventions are suggested, since they have demonstrated effectiveness in several areas [50,51]. We are also in line with previous authors when considering the potential of the web and social networks in health promotion [52], since it is the main medium where children and adolescents seek information to answer their health-related concerns [53,54]. In addition to meeting the need for health information, young consumers gain social and emotional support from peer interactions [55]. This means of communication and health promotion could be especially useful in times of a pandemic, such as the current COVID-19 pandemic, in which attendance and personal contact are forced to be reduced and telematic means become more important [56,57].

## 5. Conclusions

The results obtained with this structure confirm an adequate reliability and validity of the questionnaire. This short and easy-to-administer questionnaire will help to identify the attitudes towards healthy eating promotion of secondary school teachers, as well as assist health professionals in detecting and capturing health assets in secondary schools. Teachers with a positive attitude will be motivated to establish strategies and implement interventions to promote healthy eating in schools.

Community interventions in schools should take into account the whole educational community and be supported by instruments that measure practices and attitudes, but also knowledge. It is therefore necessary to carry out future studies with larger samples that allow deeper exploration into the subject in order to plan and implement interventions that promote greater involvement of teachers in the promotion of healthy eating in secondary schools.

The scale of attitudes towards the promotion of healthy eating among secondary school teachers is an instrument that meets the psychometric properties of reliability, internal validity, fit, as well as a good correlation, allowing its use as a research instrument in future studies.

## Figures and Tables

**Table 1:** Sociodemographic characteristics of informants.

	*N* (200)	(%)
Age		
20–30	5	2.5
30–40	61	30.5
40–50	70	35
50–60	59	29.5
+60	5	2.5
Sex		
Male	85	42.50
Female	115	57.50
Years teaching		
>10 years	151	75.5
<10 years	49	24.5
Profile		
Management team	62	31
Counsellors	17	8.5
Non-tutors	75	37.5
Tutors	46	23

**Table 2:** Descriptive data of attitude items (*n* = 200).

	Mean	*SD*	Skewness	Kurtosis
Item 1	3.02	1.065	−0.569	−1.084
Item 2	3.29	0.865	−0.823	−0.537
Item 3	3.35	0.812	−0.996	0.092
Item 4	3.41	0.732	−0.906	−0.281
Item 5	3.52	0.763	−1.541	1.655
Item 6	3.22	0.778	−0.470	−0.993
Item 7	3.17	0.905	−0.888	−0.065
Item 8	3.11	0.899	−0.712	−0.356
Item 9	3.13	0.904	−0.755	−0.327
TOTAL A	29.21	4.93	−0.0599	−0.059
CON A	16.58	3.04	−0.722	−0.252
PRO A	12.63	2.91	−0.577	−0.412

TOTAL A = Total Attitude; CON A = Concern Attitude; PRO A = Proactivity attitude.

**Table 3:** Percentages of variance explained by the common factors obtained in the factor analysis of polychoric correlations.

	Item 1	Item 2	Item 3	Item 4	Item 5	Item 6	Item 7	Item 8	Item 9
Eigenvalue	3.813	1.891	0.950	0.656	0.526	0.405	0.345	0.236	0.178
% Variance	42.365	21.016	10.559	7.286	5.843	4.501	3.833	2.622	1.976
% Cumulative Variance	4.635	63.381	73.940	81.226	87.069	91.570	95.403	98.024	100.00

**Table 4:** Factor weights: principal component analysis.

Items	PRO A (Factor 1)	CON A (Factor 2)
1	−0.72	0.713
2	−0.69	0.789
3	1.91	0.796
4	0.419	0.686
5	0.272	0.482
6	0.482	0.511
7	0.905	0.080
8	0.907	0.114
9	0.883	0.77

CON A = Concern Attitude; PRO A = Proactivity Attitude.

**Table 5:** Cronbach’s alpha and Cronbach’s alpha if the item is removed.

	Total	CON A	PRO A
Cronbach’s Alpha	0.815	0.756	0.855
If item is removed			
Item 1	0.823	0.744	
Item 2	0.809	0.691	
Item 3	0.787	0.663	
Item 4	0.781	0.698	
Item 5	0.809	0.762	
Item 6	0.790		0.913
Item 7	0.790		0.768
Item 8	0.785		0.760
Item 9	0.792		0.788

CON A = Concern Attitude; PRO A = Proactivity Attitude.

**Table 6:** Corrected item–test correlations.

	Total	CON A	PRO A
Item 1	0.345	0.475	
Item 2	0.409	0.581	
Item 3	0.592	0.665	
Item 4	0.666	0.582	
Item 5	0.396	0.369	
Item 6	0.571		0.427
Item 7	0.567		0.806
Item 8	0.604		0.824
Item 9	0.549		0.761
PRO A	0.693		
CON A	0.675		

CON A = Concern Attitude; PRO A = Proactivity Attitude.

## Data Availability

The datasets used and/or analysed during the current study are available from the corresponding author upon reasonable request.

## Abbreviations

World Health Organization (WHO); proactivity (PRO); concern (CON); Knowledge, Attitudes, Practices (KAP).
